# Incomplete Assessments: Towards a Better Understanding of Causes and Solutions. The Case of the interRAI Home Care Instrument in Belgium

**DOI:** 10.1371/journal.pone.0123760

**Published:** 2015-04-13

**Authors:** Dirk Vanneste, Johanna De Almeida Mello, Jean Macq, Chantal Van Audenhove, Anja Declercq

**Affiliations:** 1 Lucas, Center for Care Research and Consultancy, Katholieke Universiteit Leuven, Leuven, Belgium; 2 Ecole de Santé Publique, Institut de Recherche Santé et Société, Université Catholique de Louvain, Brussels, Belgium; University of Tuebingen, GERMANY

## Abstract

The chronic diseases, comorbidities and rapidly changing needs of frail older persons increase the complexity of caregiving. A comprehensive, systematic and structured collection of data on the status of the frail older person is presumed to be essential in facilitating decision-making and thus improving the quality of care provided. However, the way in which an assessment is completed has a substantial impact on the quality and value of the results. This study examines the online completion of interRAI Home Care assessments, the possible causes for incomplete assessments and the consequences of these factors with respect to the quality of care received. Our findings indicate high nurse engagement and poor physician participation. We also observed the poor completion of items in predominantly medically- oriented sections characterized by, first, the fact that the assessors felt incapable of answering certain questions, second, the absence of required data or of a competent person to fill out the data, and third, the lack of tools necessary for essential measurements. The incompleteness of assessments has a clear negative influence on outcome generation. Moreover, without the added value of support outcomes, the improvement of care quality can be impeded and information technology can easily be seen as burdensome by the assessors. We have observed that multidisciplinary cooperation is an important prerequisite to establishing high-quality assessments aimed at improving the quality of care.

## Introduction

Three decades ago, several studies identified significant and widespread poor quality of care related to the inability to identify the problems and needs of older persons [1, 2]. In 1983, Sidney Katz recognized the need for a uniform and comprehensive assessment in nursing homes [3]. All these observations were to lead to one of the cornerstones of modern geriatric care: the comprehensive geriatric assessment (CGA) [1]. A multidisciplinary, systematic and structured collection of data on the frail older person is supposed to be essential in differentiating between important and less important issues, in unraveling the complex clinical condition of a person, in guiding decision making and hence in improving healthcare processes and the quality of care provided [4–11].

Nowadays, healthcare environments are increasingly confronted with older persons characterized by chronic conditions and/or comorbidities, and in need of complex long-term care [4, 12–14]. The need to receive support from multiple service providers has significant implications for persons with complex care needs [15]. As people migrate through this maze of healthcare providers, the use of standardized, integrated, computerized and person-centered data that are available and understandable to those who must make decisions at the personal, clinical, managerial, and public policy levels has become even more fundamental in providing high-quality care. A lack of information (transfer) may result in increased assessment burden, uncoordinated care and adverse events influencing morbidity, mortality and hospital outcomes [16, 17]. Therefore, clinical information systems that typically have been designed to support single service providers in one setting no longer meet the necessary requirements [18].

The ‘first generation’ assessment instruments used collections of single-domain measures.14 Meanwhile, CGA has evolved. The interRAI suite of instruments, a ‘third generation’, multi-domain suite of compatible assessment instruments released in 2005, makes it possible to share high-quality person-centered information and to compare people, services and outcomes across settings [19–27]. This integrated system is based on:

- consistent terminology across instruments;
- a common set of ‘core’ items and definitions that are considered to be important in all care sectors (e.g., cognition, ADL) and the provision of a ‘backbone’ of critical information, ‘optional’ items and sector-specific items having identical observation timeframes and response codes—all items being classified into (care) domains referred to as ‘sections’ [14, 18];
- a common clinical assessment with an emphasis on functional assessment rather than on diagnosis;
- a common data collection method based on professional assessment skills;
- ) a common theoretical and conceptual basis providing triggers for care plans;
- algorithms generating decision support outcomes, quality improvement and monitoring measures, guidelines and care planning protocols for sectors serving similar populations;

The instruments are internationally validated, adaptable to multiple care sectors, holistic, client-centered and outcome-oriented, promote interdisciplinarity and improve continuity, efficiency and quality of care [24]. However, the interRAI assessments can only reach their full potential when computer-based information technologies are used [18, 28, 29].

A CGA being of fundamental importance [5, 8–10], the way it is handled and completed highly influences its quality and value. It is obvious that without all the required assessment data, the resulting outcome—measures, guidelines, protocols—provided to caregivers, clinicians, care managers, policymakers, researchers and other stakeholders, will invariably be limited or of poor quality [18, 22]. Therefore, our research focuses both on any sections and items that have been filled out incompletely, as well as on health professionals with a responsibility for ensuring the assessments are completed. We also discuss possible causes for incomplete assessments and consequences related to the output and care planning. To our knowledge, these aspects have never been studied before. This research will bring new insight into important facilitating and impeding conditions for performing a comprehensive assessment.

## Methods

### Context

In Belgium, the interRAI assessment instruments were adapted to the Belgian healthcare context, and a web application (hereafter referred to as BelRAI) was developed to support the use of the assessments in Belgium’s three official languages: Flemish-Dutch, Walloon-French and German [30–37]. Usability studies show that BelRAI allows caregivers to assess the condition of a frail older person in a multidisciplinary way and to exchange person-centered information over time and between different care providers, safely, anywhere and at any time. The whole system was developed in collaboration with prospective users and stakeholders [38]. Online, the health professional responsible for the completion of the assessment can invite each professional involved in the care for the older person to complete the section(s) of the assessment related to his or her area of expertise. The system reveals conflicting answers and uses an interdependency system with data checks, validations and restrictions in order to prevent users filling out erroneous, inappropriate or inconsistent information and to draw attention to dubious answers. An online support platform—BelRAIWiki—offers ‘one click away’ background information in order to facilitate the assessment procedure and enhance the involvement and training of professionals from various disciplines and healthcare sectors.

In principle, assessments should always be filled out completely (100%). The software used should be programmed in a way that users are obliged to answer all questions. However, due to unavoidable circumstances, this feature was temporarily turned off in the BelRAI software and users were told the assessment should be at least 75% complete. This is intended only as a temporary measure. However, the current situation has made it possible to study which items are most often left blank once the opportunity to do so is created. This kind of knowledge allows for the targeting of specific coding problems during training, not only in Belgium, but in any country where the interRAI instruments are used.

### Participants

The participants in the study were health professionals (nurses, occupational therapists, social workers, psychologists, physiotherapists, speech therapists, and physicians) caring for older persons—clients—in home care projects [39]. These professionals underwent a two-day training course and a follow-up training course lasting one day on how to fill out an interRAI HC assessment using the BelRAI web application (http://www.belrai.org). The clients were at least 65 years old, frail and eligible to be admitted into a nursing home.

### Data collection

Every interRAI HC instrument is filled out upon the inclusion of the frail older person in the home care projects (baseline), based on observation, shared data, and using data obtained by interviewing the older person and the main informal caregiver. While several health professionals of different disciplines could participate in the same assessment, one health professional was *responsible* for ensuring the completion of the assessment. In this study, we used the data related to the ‘*responsible*’ health professionals.

### Ethical considerations

BelRAI meets the privacy standards of the Sectoral Committee of the Commission for the Protection of Privacy in Belgium [40]. Furthermore, the study was approved by the same Belgian Privacy Commission and by the Ethics committee of the Belgian universities Université Catholique de Louvain and KU Leuven (B40320108337). A formal procedure was implemented in order to make sure that caregivers could fill out the questionnaires on a secured website [41]. Frail older persons were asked to sign an informed consent agreement. In cases where these persons or clients were not capable of signing this document, a family member or another legal representative signed it on their behalf, as stipulated by Belgian law. Clients were able to withdraw their participation at any time, without any consequences for the care they received. All data were anonymized before the dataset was sent to the researchers for analysis.

### Data analysis

All data were derived from first assessments that were at least 75% complete (see above). This arbitrary cut-off was determined at the start of the project for practical and policy reasons. It was reasoned that if a caregiver really intends to use the assessment outcomes, he or she would complete at least 75% of the assessment. An assessment completed for less than 75% lacks sufficient information for the generation of any meaningful output.

As the use of free input fields or text boxes is not required to calculate outcomes, we did not include data related to items such as other diagnoses (I2)—name and International Classification of Disease code—and medication (M1)—name, dose, unit, administration, frequency, pro re nata (PRN), and drug identification number—in our study. Nor did we take into account:

- ‘administrative’ items such as name (A1a-d), gender (A2), date of birth (A3), marital status (A4), personal identification numbers (A5a), other payment categories (A7k-m), reason for assessment (A8), postal code (A10), substitute decision maker (A18d), treating doctor (A20), education (A22), ethnicity/race/nationality (B3a-g), primary language (B4), last day of stay (T1), living status after discharge (T2), signature (U1) and date (U2);
- the item indicating recent falls (J12) since it is only assessed during follow-up assessments and not during the first assessment;
- the item indicating physical restraint (N4) since it is replaced by full bed rails (N6a), trunk restraint (N6b), and chair prevents rising (N6c) in the BelRAI web application;
- the item indicating the second informal helper (P1a2, P1b2, P1c2 and P1d2) as most clients in the home care projects do not have a second informal caregiver;
- the items R3, R4 and R5 as these are not assessed if the client did not deteriorate in last 90 days—to gather information on the overall completion score of this section we focused on data relating to care goals met (R1) and self-sufficiency change (R2).

Data analysis was performed in two steps. First, descriptive statistics were calculated to determine to what extent each of the items of the interRAI HC instrument was completed and, second, to see which type of health professional was responsible for the completeness of the assessment. Statistical analysis was performed using STATA 11.1 (StataCorp, College Station, Texas).

## Results

From March 2010 until January 2013, 5,117 assessments were completed for at least 75%. The following research is based on data originating from these assessments.

Table 1 shows high completion scores for assessment items regarding Section A—Identification information (≥98.84%), Section B—Intake and initial history (≥98.48%), Section C—Cognition (≥99.18%), Section D—Communication and vision (≥99.43%), Section E—Mood and behavior (≥98.12%), Section F—Psychosocial well-being (≥99.18%), and Section H—Continence (≥99.18%). In Section J—Health conditions—all items have a score between 96.74% (gastrointestinal/genitourinary bleeding) and 99.41% (tobacco). Also, Section L—Skin condition (≥98.42%; L7 = 97.97%) and Section P—Social supports (≥98.23%) have high completion percentages. Most items of Section Q—Environmental assessment have high completion scores (≥98.07%; Q3b, Q3C, and Q4 ≥97.52%).

**Table 1:** Description of Sections, Completion of Items and Affected Outcomes.

Generic Variable Name^a^	Section Names and Items	Completion %	95% CI	Affected Outcomes	
				Clinical Assessment Protocols	Scales & Screening Algorithms
**Section A**	**Identification information**				
A9	Reference date	99.73	0.996–0.999		AGE
A11b	Residential/Living status	98.84	0.986–0.991		
A12a	Living arrangement	99.26	0.990–0.995	BRITSU	
A12b	Lives with someone new	98.92	0.986–0.992		
A12c	Better living elsewhere	99.37	0.992–0.996	ABUSE	
A13	Time since last hospital stay	99.10	0.988–0.994		
**Section B**	**Intake and initial history**				
B2	Date case opened	99.41	0.938–0.950		
B5a	History: LTCF	98.93	0.986–0.992	RISK	MAPLe
B5b	History: Board and care home, assisted living	98.85	0.986–0.991		
B5c	History: Psychiatric hospital	98.48	0.981–0.988		
B5e	History: Mental health residence	98.81	0.985–0.991		
**Section C**	**Cognition**				
C1	Daily decision-making	99.80	0.997–0.999	RISK, RESTR, COMMUN, FEEDTB, URIN, BOWEL, IADL,	MAPLe, CPS2, RUGs
				ADL, COGNIT, SOCFUNC	
C2a	Short-term memory	99.57	0.993–0.997	RISK, IADL, ADL, COGNIT, SOCFUNC	MAPLe, CPS2, RUGs
C2b	Procedural memory	99.30	0.990–0.995	IADL, ADL, COGNIT, SOCFUNC	CPS2, RUGs
C2c	Situational memory	99.41	0.992–0996		
C3a	Easily distracted	99.18	0.989–0.994	ADL, COGNIT, DELIR, DEHYD, BOWEL	
C3b	Disorganized speech	99.26	0.990–0.995	COGNIT, DELIR, DEHYD, BOWEL	
C3c	Mental function varies over day	99.37	0.992–0.996	ADL, COGNIT, DELIR, DEHYD, BOWEL	
C4	Acute change in mental status	99.26	0.990–0.995	ADL, COGNIT, DELIR, DEHYD, BOWEL	
C5	Change in decision-making	99.32	0.991–0.995	ADL, COGNIT	MAPLe, CHESS
**Section D**	**Communication and vision**				
D1	Making self understood	99.63	0.995–0.998	RISK, COGNIT, COMMUN, IADL, ADL, SOCFUNC	MAPLe, CHESS, COMM, RUGs
D2	Ability to understand others	99.65	0.995–0.998	RISK, COGNIT, COMMUN, SOCFUNC	MAPLe, COMM
D3a	Hearing	99.43	0.992–0.996		
D4a	Vision	99.53	0.993–0.997		
**Section E**	**Mood and behavior**				
E1a	Negative statements	99.63	0.995–0.998	ABUSE, ENVIR, MOOD	RUGs, DRS
E1b	Anger	99.49	0.993–0.997	ABUSE, ENVIR, MOOD	RUGs, DRS
E1c	Unrealistic fears	99.53	0.993–0.997	ABUSE, ENVIR, MOOD	RUGs, DRS
E1d	Repetitive health complaints	99.32	0.991–0.995	ABUSE, ENVIR, MOOD	RUGs, DRS
E1e	Anxious complaints	99.39	0.992–0.996	ABUSE, COGNIT, ENVIR, MOOD	RUGs, DRS
E1f	Facial expressions	99.43	0.992–0.996	ABUSE, ENVIR, MOOD	RUGs, DRS
E1g	Crying	99.12	0.989–0.994	ABUSE, ENVIR, MOOD	RUGs, DRS
E1h	Recurrent statements	99.32	0.991–0.995	COGNIT	
E1i	Withdrawal	99.39	0.992–0.996	ABUSE	
E1j	Reduced social interactions	99.16	0.989–0.994	ABUSE	
E1k	Lack of pleasure	99.24	0.990–0.995		
E2a	Self-report: Little interest	98.22	0.979–0.986		
E2b	Self-report: Anxious, restless, uneasy	98.18	0.978–0.985		
E2c	Self-report: Sad, depressed, hopeless	98.12	0.978–0.985		
E3a	Wandering	99.53	0.993–0.997	RISK, COGNIT, BEHAV	MAPLe, RUGs
E3b	Verbal abuse	99.53	0.993–0.997	RISK, BEHAV	MAPLe, RUGs
E3c	Physical abuse	99.57	0.994–0.997	RISK, COGNIT, BEHAV	MAPLe, RUGs
E3d	Socially inappropriate behavior	99.57	0.994–0.997	RISK, BEHAV	MAPLe, RUGs
E3e	Resists care	99.63	0.995–0.998	RISK, BEHAV	MAPLe, RUGs
E3f	Inappropriate sexual behavior	99.53	0.993–0.997	RISK, BEHAV	MAPLe, RUGs
**Section F**	**Psychosocial well-being**				
F1a	Social activities	99.63	0.995–0.998		
F1b	Visit by relation or family member	99.57	0.994–0.997		
F1c	Other interaction with relation or family member	99.65	0.995–0.998		
F1d	Lonely	99.27	0.990–0.995	SOCFUNC	
F1e	Openly expresses conflict with family	99.36	0.991–0.996	ABUSE	
F1f	Fearful of family member	99.53	0.993–0.997	ABUSE	
F1g	Neglected or abused	99.55	0.994–0.997	ABUSE	
F2	Change in social activities	99.18	0.989–0.994	SOCFUNC	
F3	Length of time alone	99.53	0.993–0.997	BRITSU, SOCFUNC	
F4	Major life stressors	99.45	0.993–0.997		
**Section G**	**Functional status**				
G1aa	Meal preparation—performance	99.28	0.990–0.995		RUGs, IADLP
G1ab	Meal preparation—capacity	95.60	0.950–0.962	BRITSU, IADL	MAPLe, IADLC
G1ba	Ordinary housework—performance	99.36	0.991–0.996		IADLP
G1bb	Ordinary housework—capacity	95.62	0.951–0.962	BRITSU, IADL	MAPLe, IADLC
G1ca	Managing finances—performance	99.37	0.992–0.996		IADLP
G1cb	Managing finances—capacity	95.66	0.951–0.962		IADLC
G1da	Managing medications—performance	99.43	0.992–0.996		RUGs, IADLP
G1db	Managing medications—capacity	95.88	0.953–0.964		MAPLe, IADLC
G1ea	Phone use—performance	99.37	0.992–0.996		RUGs, IADLP
G1eb	Phone use—capacity	94.72	0.941–0.953		IADLC
G1fa	Stairs—performance	99.26	0.990–0.995	PACTIV	IADLP
G1fb	Stairs—capacity	91.38	0.906–0.922	ENVIR	IADLC
G1ga	Shopping—performance	99.45	0.993–0.997		IADLP
G1gb	Shopping—capacity	94.45	0.938–0.951	BRITSU, IADL	IADLC
G1ha	Transportation—performance	99.14	0.989–0.994		IADLP
G1hb	Transportation—capacity	91.89	0.911–0.926	BRITSU, IADL	MAPLe, IADLC
G2a	Bathing—performance	98.96	0.987–0.992		MAPLe
G2b	Personal hygiene—performance	99.22	0.990–0.995	RISK, RESTR, IADL, ADL	MAPLe, ADLH
G2c	Dressing upper body—performance	99.12	0.989–0.994		
G2d	Dressing lower body—performance	99.20	0.990–0.994		
G2e	Walking—performance	98.60	0.983–0.989	URIN	PURS
G2f	Locomotion—performance	99.14	0.989–0.994	RISK, RESTR, IADL, ADL, PACTIV	MAPLe, ADLH
G2g	Transfer toilet—performance	98.67	0.984–0.990	RISK, PULCER	MAPLe, RUGs
G2h	Toilet use—performance	99.04	0.988–0.993	RESTR, BOWEL, IADL, ADL	MAPLe, RUGs, ADLH
G2i	Bed mobility—performance	98.93	0.986–0.992	BOWEL, PULCER	RUGs, PURS
G2j	Eating—performance	99.57	0.994–0.997	RESTR, BOWEL, IADL, ADL, COGNIT, SOCFUNC	MAPLe, CPS2, RUGs, ADLH
G3	Primary mode of locomotion	99.22	0.990–0.995	RISK	MAPLe
G4	Distance walked	97.28	0.968–0.977		
G5	Distance wheeled self	97.79	0.974–0.982		
G6a	Hours of exercise or physical activity	99.14	0.989–0.994	PACTIV, ENVIR	MAPLe
G6b	Days went out	99.14	0.989–0.994	RISK	MAPLe
G7a	Person believes can improve	98.48	0.981–0.988	IADL, PACTIV	
G7b	Caregiver believes person can improve	97.91	0.975–0983	BOWEL, IADL, PACTIV	
G8a	Change in ADL status	99.10	0.988–0.994	RISK, URIN, IADL, ADL	MAPLe, CHESS
G9a	Drove car	99.16	0.989–0.994		
G9b	Suggestion to limit driving	98.55	0.982–0.989		
G12	Timed 4-meter walk	87.63	0.867–0.885		
**Section H**	**Continence**				
H1	Bladder continence	99.36	0.991–0.996	RISK, URIN, BOWEL, PULCER	MAPLe
H2	Urinary collection device	99.18	0.989–0.994	URIN, PULCER	
H3	Bowel continence	99.39	0.992–0.996	BOWEL	PURS
H4	Pads worn	99.36	0.991–0.996		
**Section I**	**Disease diagnoses**				
I1a	Hip fracture	93.77	0.931–0.944	URIN, BOWEL, ADL	
I1b	Other fracture	93.64	0.930–0.943		
I1c	Alzheimer’s disease	91.18	0.910–0.925	RISK, COGNIT	MAPLe
I1d	Other dementia	91.50	0.907–0.923	COGNIT	
I1e	Hemiplegia	92.03	0.913–0.928		RUGs
I1f	Multiple sclerosis	92.36	0.916–0.931		RUGs
I1g	Paraplegia	91.97	0.912–0.927		
I1h	Parkinson's disease	91.67	0.909–0.924		
I1i	Quadriplegia	91.79	0.910–0.925	RESTR	RUGs
I1j	Stroke	91.01	0.902–0.918		
I1k	Coronary heart disease	89.08	0.882–0.899		
I1l	Congestive heart failure	88.89	0.880–0.897		
I1m	Chronic obstructive pulmonary disease	89.80	0.890–0.906		
I1n	Anxiety	91.40	0.906–0.922		
I1o	Depression	90.72	0.899–0.915		
I1p	Schizophrenia	90.91	0.901–0.917		
I1q	Pneumonia	91.85	0.911–0.926	URIN, BOWEL, ADL	RUGs
I1r	Urinary tract infection	91.64	0.909–0.924		
I1s	Cancer	92.09	0.913–0.928		
I1t	Diabetes mellitus	91.93	0.912–0.927		RUGs
I1w	Bipolar disorder	90.74	0.899–0.915		
**Section J**	**Health conditions**				
J1	Falls	98.96	0.987–0.992	RISK, ADL, FALLS	MAPLe
J2a	Difficulty standing	98.96	0.987–0.992		
J2b	Difficulty turning around	98.87	0.986–0.992		
J2c	Dizziness	98.70	0.984–0.990	DEHYD, CARDIO, DRUG	
J2d	Unsteady gait	98.81	0.985–0.991	ENVIR	
J2e	Chest pain	98.50	0.982–0.988	CARDIO, DRUG	
J2f	Difficulty clearing airway	98.28	0.979–0.986		
J2g	Abnormal thought process	98.59	0.983–0.989	ENVIR	
J2h	Delusions	98.69	0.984–0.990	ENVIR	RUGs
J2i	Hallucinations	98.57	0.982–0.989	ENVIR	RUGs
J2j	Aphasia	97.91	0.975–0.983		RUGs
J2k	Constipation	98.36	0.980–0.987	DEHYD	
J2l	Diarrhea	98.34	0.980–0.987	URIN, DEHYD	
J2m	Acid reflux	98.32	0.980–0.987		
J2n	Vomiting	97.91	0.975–0.983	DEHYD	RUGs, CHESS
J2o	Difficulty falling asleep	98.46	0.981–0.988		
J2p	Too much sleep	98.44	0.981–0.988		
J2q	Fever	98.38	0.980–0.987	DEHYD	RUGs
J2r	Gastrointestinal/Genitourinary bleeding	96.74	0.962–0.972		RUGs
J2s	Peripheral edema	97.81	0.974–0.982	DRUG	CHESS
J2t	Aspiration	98.53	0.982–0.989		
J2mm	Poor hygiene	98.53	0.982–0.989	ABUSE	
J3	Dyspnea	97.62	0.972–0.980	CARDIO, DRUG	CHESS, PURS
J4	Fatigue	98.51	0.982–0.988		
J5a	Pain frequency	98.75	0.984–0.991	PAIN	PURS, PAIN
J5b	Pain intensity	97.48	0.970–0.979	PAIN	PAIN
J5c	Pain consistency	97.17	0.967–0.976		
J5d	Breakthrough pain	97.46	0.970–0.979		
J5e	Pain control	97.69	0.973–0.981		
J6a	Unstable conditions	98.71	0.984–0.990	ABUSE, ENVIR	
J6b	Flare-up	98.67	0.984–0.990	ADL	
J6c	End-stage disease	98.55	0.982–0.989	ADL, COGNIT, NUTR	RUGs, CHESS
J7	Self-rated health	98.65	0.983–0.990	ABUSE, ENVIR, DRUG	
J8a	Tobacco	99.41	0.992–0.996	ADD	
J8b	Alcohol	98.92	0.986–0.992	ADD	
**Section K**	**Oral and nutritional status**				
K1ab	Height—cm	80.55	0.795–0.816	ABUSE, NUTR	BMI
K1bb	Weight—kilograms	81.16	0.801–0.822	ABUSE, NUTR	BMI
K2a	Weight loss	98.30	0.979–0.987	ABUSE, DEHYD	RUGs, CHESS, PURS
K2b	Fluid intake	97.56	0.971–0.980	ABUSE, DEHYD	
K2c	Dehydrated	97.58	0.972–0.980	DEHYD	RUGs, CHESS
K2h	Fluid output exceeds input	97.30	0.969–0.977		
K3	Mode of nutritional intake	98.87	0.986–0.992	FEEDTB	MAPLe, RUGs
K4a	Dentures	96.66	0.962–0.972		
K4b	Broken teeth	96.70	0.962–0.972		
K4c	Difficulty chewing	97.42	0.970–0.979		
K4d	Dry mouth	97.17	0.967–0.976		
**Section L**	**Skin condition**				
L1	Most severe pressure ulcer	98.83	0.985–0.991	PULCER	MAPLe, RUGs
L2	Prior pressure ulcer	98.42	0.981–0.988	PULCER	PURS
L3	Other skin ulcer	98.44	0.981–0.988	PULCER	
L4	Major skin problems	98.44	0.981–0.988		RUGs
L5	Skin tears or cuts	98.48	0.981–0.988		RUGs
L6	Other skin condition or changes	98.46	0.981–0.988		RUGs
L7	Foot problems	97.97	0.976–0.984		
**Section M**	**Medication**				
M2	Drug allergy	89.49	0.886–0.903		
M3	Drug adherence	90.76	0.900–0.916	ABUSE	
**Section N**	**Treatments and procedures**				
N1a	Influenza vaccine	92.34	0.916–0.931		
N1b	Pneumovax vaccine	88.61	0.877–0.895		
N1c	Mammogram	91.01^b^	0.901–0.920		
N1d	Blood pressure	94.68	0.941–0.953		
N1e	Dental exam	91.89	0.911–0.926		
N1f	Hearing exam	91.78	0.910–0.925		
N1g	Eye exam	92.16	0.914–0.929		
N1h	Colonoscopy	92.28	0.915–0.930		
N2a	Chemotherapy	96.15	0.956–0.967		RUGs
N2b	Dialysis	95.84	0.953–0.964		RUGs
N2c	Infection control segregation	96.03	0.955–0.966		
N2d	IV medication	95.97	0.954–0.965		RUGs
N2e	Oxygen therapy	96.13	0.956–0.967		RUGs
N2f	Radiation	96.97	0.954–0.965		RUGs
N2g	Suctioning	96.03	0.955–0.966		RUGs
N2h	Tracheostomy care	95.97	0.954–0.965		RUGs
N2i	Transfusion	95.92	0.954–0.965		RUGs
N2j	Ventilator or respirator	95.94	0.954–0.965		RUGs
N2k	Wound care	95.80	0.952–0.963	PULCER	RUGs
N2l	Scheduled toileting program	95.37	0.948–0.959	URIN	
N2m	Palliative care program	95.18	0.946–0.958		
N2n	Turning/Repositioning program	95.35	0.948–0.959		RUGs
N3aa	Home health aides—days	94.27	0.936–0.949		
N3ab	Home health aides—minutes	58.00	0.566–0.594		
N3ba	Home nurse—days	96.42	0.959–0.969		
N3bb	Home nurse—minutes	69.14	0.679–0.704		
N3ca	Homemaking services—days	93.57	0.929–0.942		
N3cb	Homemaking services—minutes	58.43	0.571–0.598		
N3da	Meals—days	92.10	0.914–0.928		
N3ea	Physical therapy—days	92.09	0.913–0.928	ADL	RUGs
N3eb	Physical therapy—minutes	50.44	0.491–0.518		RUGs
N3fa	Occupational therapy—days	90.13	0.893–0.909		RUGs
N3fb	Occupational therapy—minutes	39.73	0.384–0.411		RUGs
N3ga	Speech therapy—days	89.99	0.892–0.908		RUGs
N3gb	Speech therapy—minutes	37.97	0.366–0.393		RUGs
N3ha	Psychological therapy—days	89.88	0.891–0.907		
N3hb	Psychological therapy—minutes	38.21	0.369–0.395		
N5a	Overnight hospital stay	94.61	0.940–0.952	ADL	
N5b	Emergency room visit	93.14	0.924–0.938		
N5c	Physician visit—90 day	92.75	0.920–0.935		
N6a	Full bed rails	98.48	0.981–0.988		
N6b	Trunk restraint	98.46	0.981–0.988	RESTR	
N6c	Chair prevents rising	98.28	0.979–0.986	RESTR	
**Section O**	**Responsibility**				
O1a	Legal guardian	95.97	0.954–0.965		
**Section P**	**Social supports**				
P1a1	Informal help-relationship—1	99.74	0.996–0.999	BRITSU	
P1b1	Lives with person—1	98.94	0.986–0.992		
P1c1	IADL care—1	99.13	0.988–0.994		
P1d1	ADL care—1	99.10	0.988–0.994		
P2a	Unable to continue informal care	98.75	0.984–0.991		
P2b	Informal helper stress	98.61	0.983–0.990	ABUSE	
P2c	Family overwhelmed	98.23	0.978–0.986		
P4	Strong and supportive relationship with family	98.30	0.979–0.987		
**Section Q**	**Environmental assessment**				
Q1a	Disrepair of the home	98.81	0.985–0.991	ENVIR	MAPLe
Q1b	Squalid conditions	98.59	0.983–0.989	ENVIR	MAPLe
Q1c	Inadequate heating or cooling	98.57	0.982–0.989	ENVIR	MAPLe
Q1d	Lack of personal safety	98.26	0.979–0.986		MAPLe
Q1e	Limited access to home or rooms	98.50	0.982–0.988	ENVIR	MAPLe
Q2	Handicapped re-engineered apartment	98.07	0.977–0.984		
Q3a	Availability of emergency assistance	98.08	0.977–0.985		
Q3b	Accessibility to grocery store	97.52	0.971–0.979		
Q3c	Availability of home delivery of groceries	97.52	0.971–0.979		
Q4	Trade-offs	97.99	0.976–0.984		
**Section R**	**Discharge potential and overall status**				
R1	Care goals met	41.88	0.405–0.432		
R2	Self-sufficiency change	67.91	0.666–0.692	BOWEL, ADL, COGNIT, DRUG	
R3	Independent ADL areas	34.45^c^	0.332–0.358		
R4	Independent IADL areas	34.53^c^	0.332–0.358		
R5	Onset of precipitating event	35.96^c^	0.337–0.363		

Clinical Assessment Protocols (CAPs): BRITSU = Brittle Support, ABUSE = Abusive Relationship, RISK = Institutional Risk, RESTR = Physical Restraints, COMMUN = Communication, FEEDTB = Feeding Tube, URIN = Urinary Incontinence, BOWEL = Bowel Conditions, IADL = Instrumental Activities of Daily Living, ADL = Activities of Daily Living, COGNIT = Cognitive Loss, SOCFUNC = Social Relationship, DELIR = Delirium, DEHYD = Dehydration, ENVIR = Home Environment Optimization, MOOD = Mood, BEHAV = Behavior, PACTIV = Physical Activities Promotion, PULCER = Pressure Ulcer, FALLS = Falls, CARDIO = Cardio-Respiratory Conditions, DRUG = Medications, PAIN = Pain, NUTR = Undernutrition, ADD = Addict. Scales and Screening Algorithms: AGE = Age Years Scale, MAPLe = Method for Assigning Priority Levels, CPS2 = Cognitive Performance Scale 2, RUGs = Resource Utilization Groups, CHESS = Changes in Health, End-Stage Disease, Signs, and Symptoms Scale, COMM = Communication Scale, DRS = Depression Rating Scale, ADLH = Activities of Daily Living Hierarchy, IADLC/P = Instrumental Activities of Daily Living Capacity/Performance, PURS = Pressure Ulcer Risk Scale, PAIN = Pain, BMI = Body Mass Index.

aiCode

bCorrected for only females.

cItems assessed in cases of deterioration of the client in last 90 days (Item R2).

Lower completion scores are shown in items of Section G—Functional status, Section I—Disease diagnoses, Section K—Oral and nutritional status, Section M—Medications, Section, N—Treatment and procedures, Section O—Responsibility, and Section R—Discharge potential and overall status. To gain more insight into the completion of these sections, we address the completion scores of the individual items.

Particularly in Section G—Functional status—lower completion percentages are seen for the IADL *capacity* items of meal preparation (95.60%), ordinary housework (95.62%), managing finances (95.66%), managing medications (95.88%), phone use (94.72%), stairs (91.38%), shopping (94.45%) and transportation (91.89%). On the other hand, the IADL *performance* items score higher completion percentages (≥99.14%). While high scores are shown for ADL and the other items, we observe a lower completion score for the timed 4- meter walk item (87.63%).

For all the items of Section I—Disease diagnoses—we note a lower completion percentage between 88.89% (congestive heart failure) and 93.77% (hip fracture).

In Section K—Oral and nutritional status—the items height and weight have low completion percentages of 80.55% and 81.16%, respectively. The other items score between 96.66% (dentures) and 98.87% (mode of nutritional intake).

We observe a low score in Section M—Medications with item completion rates of 89.49% (drug allergy) and 90.76% (drug adherence).

In Section N—Treatment and procedures—the completion of the observed *minutes* for home health aides (58.00%), home nurse (69.14%), homemaking services (58.43%), physical therapy (50.44%), occupational therapy (39.73%), speech therapy (37.97%) and psychological therapy (38.21%) is very low. Other low completion scores are 92.34% (influenza vaccine), 88.61% (pneumovax vaccine), 91.01% (mammogram, corrected for only females), 94.68% (blood pressure), 91.89% (dental exam), 91.78% (hearing exam), 92.16% (eye exam), 92.28% (colonoscopy), 94.27% (home health aides/days), 93.57% (homemaking services/days), 92.10% (meals/days), 92.09% (physical therapy/days), 90.13% (occupational therapy/days), 89.99% (speech therapy/days), 89.88% (psychological therapy/days), 94.61% (overnight hospital stay), 93.14% (emergency room visit) and 92.75% (physician visit/90 day). Completion scores between 95.18% and 96.97% are shown for chemotherapy, dialysis, infection control segregation, IV medication, oxygen therapy, radiation, suctioning, tracheostomy care, transfusion, ventilator or respirator, wound care, scheduled toileting program, palliative care program, turning/repositioning program, and home nurse/days. However, full bed rails, trunk restraint and chair prevents rising have scores between 98.28% and 98.48%.

In Section O—Responsibility—we note a completion score of 95.97% for the item legal guardian.

The two first items, care goals met and self-sufficiency change, of Section R—Discharge potential and overall status—show a completion score of 41.88% and 67.91%, respectively. In cases of deterioration of the client in last 90 days (R2 code = 2), independent ADL areas and independent IADL areas score 34.45% and 34.53%, onset of precipitating event scores 34.53%.

Health professionals of different disciplines, nurses (62.18%), occupational therapists (21.46%), social workers (9.87%), psychologists (4.77%), physiotherapists (1.43%), speech therapists (0.28%), and physicians (0.02%) ensured the completion of 5,117 questionnaires in total (Table 2).

**Table 2:** ‘Responsible’ Health Professionals.

‘Responsible’ Health Professionals^a^	Proportion % (N = 5,117)	95% CI
Nurses	62.18	0.6086–0.6351
Occupational therapists	21.46	0.2033–0.2258
Social workers	9.87	0.0905–0.1069
Psychologists	4.77	0.0418–0.0535
Physiotherapists	1.43	0.0110–0.0175
Speech therapists	0.28	0.0013–0.0042
Physicians	0.02	-0.0002–0.0006

CI = confidence interval

aThese health professionals have assumed *responsibility* for ensuring the completion of the assessments.

## Discussion

### Possible causes of incomplete assessments

Based on our data, individual items in several sections of the interRAI HC assessment instrument have lower completion scores. Possible causes can be found in the fact that first, the assessors felt incapable of answering certain questions, second, the absence of required data or a competent person, and third, the insufficient presence of tools necessary for carrying out essential measurements.

The assessment of the functional status of the client seems to be more demanding. Items concerning IADL capacity—Section G—were completed less well. These items require thorough observation and thinking by the assessor with regard to the frail older person’s presumed ability to carry out an activity [27]. In the home care sector, where contact with clients tends to be shorter than in the institutional care sector and where observation is more difficult to put into practice, this may be less evident [37]. Due to the fact that the data comes from baseline assessments, many were performed during the first visit of the caregiver in the clients’ home. Caregivers can perhaps not observe the client during a sufficient period of time and base their assessment on the interview with the client and informal caregiver. Other reasons may be that health professionals (for example, newcomers) have received inadequate training to perform assessments, that they receive insufficient information from other caregivers, or lack the time required to assess the situation correctly. Continuing education and training programs concerning the theoretical and practical aspects of the assessment instrument can contribute to a more successful completion of these and other sections. For home care organizations which are more fragmented and diverse, these training sessions are also a good opportunity to enhance communication and collaboration [38]. In addition to this, a significant expenditure of resources with regard to adequate staffing in healthcare environments and enough available time in view of performing assessments is a major advantage. It is possible that the Section R items—Discharge potential and overall status—have been completed less well for the same reasons.

Sections dealing with mainly medically-oriented data, including disease diagnoses (Section I), drug allergy and adherence (Section M), and (preventive) treatments and procedures (Section N) exhibit (completion) deficits. Table 2 shows that nurses play a leading role in checking, initiating and inviting other caregivers to help complete, validate, and finalize a client’s interRAI HC assessment. This is less the case for occupational therapists, social workers, psychologists, physiotherapists and speech therapists. Physicians occasionally assist in the completion of the questionnaires but rarely (0.02%) do they assume the responsibility for ensuring the completion of the assessment. It seems possible that medically- oriented sections are less thoroughly completed because in a home care situation non- physicians do not always have access to the necessary medical information. In our view, it is essential that physicians are motivated to cooperate and to share crucial information.

The assessment of the timed 4-meter walk (Section G) is intended to record an objective benchmark for comparison of the client’s performance upon subsequent reassessments. The assessment of client’s current weight and height (Section K) allows for the monitoring of nutrition, hydration status, and weight stability over time. Items concerning services and therapies (Section N) require the recording of the duration of these activities of minutes. These measurements need calibrated tools such as a stopwatch, scale, and measuring device. Perhaps this is a problem in the home care sector, since these sections also have a low percentage of completion.

### Consequences of incomplete assessments

A comprehensive, systematic and structured collection of data of the frail older person is presumed to be essential in improving the quality of care [4, 6, 7]. Assessments are of fundamental importance but the usefulness and value of such assessments is closely linked to any decision-making or interventions that result from the assessments [1]. Furthermore, the use of such an instrument very much determines the quality of the assessment. It is obvious that without the required data, the guidelines and care planning protocols, decision support outcomes, and quality improvement and monitoring measures cannot be calculated. The absence of outcomes may complicate the care planning process and even prevent the improvement of care quality. Also, the assessment process can easily be seen as additional work.

InterRAI Clinical Assessment Protocols (CAPs) [21, 42] are designed to assist caregivers in interpreting all the assessed information. They help to determine risk or priority areas for care. In the next to right-most column in Table 1 we indicate the affected CAPs in the case of missing or incomplete information. The right-most column in the same Table shows the affected interRAI scales, status and outcome measures [43–45], case-mix classification [46, 47], and screening algorithms [48]. For instance, if information about meal preparation—capacity—(Section G) is insufficient, then calculation of the Instrumental Activities of Daily Living (IADL) CAP, Brittle Support (BRITSU) CAP, Instrumental Activities of Daily Living Capacity (IADLC) scale, and Method for Assigning Priority Levels (MAPLe) will be impossible. Data on stairs—performance—, locomotion—performance—, hours of exercise or physical activity, person believes can improve, and caregiver believes can improve, are needed to calculate the Physical Activities Promotion (PACTIV) CAP. If information about hip fracture (Section I) is insufficient, then calculation of the Urinary Incontinence (URIN) CAP, Bowel Conditions (BOWEL) CAP, and Activities of Daily Living (ADL) CAP will be impossible. Information about height and weight (Section K) is needed to calculate the BMI.

## Limitations

First, the sample is not representative for all older people living at home because clients were recruited at the time of entry into the home care projects. Second, each project is evaluated (amongst other factors) based on the assessment outcomes, which may influence the way in which the assessors completed the assessments. Third, as we are dealing with projects, the assessors may have known the clients for only a short period of time, and thus insufficiently.

## Conclusions

When a CGA is completed in a coordinated and multidisciplinary way, whereby the items are filled out by all involved health professionals on the basis of their expertise or experience, we can assume that the assessment reflects the real situation of the client. In this way, the assessment can meet the objective of developing an overall care plan and ensuring long-term follow-up. Without the required data on record, outcomes cannot be calculated and it must be clear that an incomplete assessment cannot fully contribute to improvements in diagnostic accuracy, care optimization and quality of care. Moreover, incomplete assessments may result in uncoordinated care and subsequent adverse events.

Multidisciplinarity is an important precondition for establishing high-quality assessments and related outcomes that offer more insight into the complexity of the healthcare process and a higher quality of care. Ignorance of the rationale of a multidimensional assessment system and process can impede caregivers in cooperating or induce resistance to change [49]. By contrast, a good understanding of such tools and systems can prevent them being seen as unnecessarily burdensome, as opposed to an integral part of the decision- making process [4]. Health professionals, including physicians and managers should be convinced that the use and full completion of a comprehensive information system contributes to integrated quality care. It is important to continuously inform the intended users of the benefits and to motivate all stakeholders to increase their involvement and collaboration [29, 38]. This is certainly the case in a more fragmented home care sector, where information technology presents a significant opportunity to upgrade the existing communication strategy.

It seems also appropriate that extra attention should be paid to these theoretical and practical aspects of the assessment process during the education and training of health professionals and to the allocation of the necessary resources.
